# Study Protocol of a Distance Learning Intervention to Support Social Emotional Learning and Identity Development for Adolescents Using Interactive Mobile Technology

**DOI:** 10.3389/fpubh.2021.623283

**Published:** 2021-01-28

**Authors:** Megan Cherewick, Sarah Lebu, Christine Su, Lisa Richards, Prosper F. Njau, Ronald E. Dahl

**Affiliations:** ^1^Department of Health Sciences, California State University East Bay, Hayward, CA, United States; ^2^Institute of Human Development, University of California Berkeley, Berkeley, CA, United States; ^3^Health for a Prosperous Nation, Dar es Salaam, Tanzania

**Keywords:** developmental science, adolescence, learning with technology, social emotional learning, identity development, distance learning

## Abstract

**Background:** The maturational period from age 10 to 14—often referred to as very young adolescents (VYAs)—represents a dynamic period of learning and neurobehavioral development as individuals transition from childhood to adolescence. This developmental period presents a window of opportunity for strategic investment to improve trajectories of health, education and well-being among young people. More specifically, neurodevelopmental changes during pubertal maturation influence neural circuitry involved in processing emotions, risks, rewards and social relationships. Technology can be leveraged to create social emotional learning experiences for VYAs and provide opportunities for flexible, distance learning in low-income countries. The aim of this study protocol is to detail how insights from developmental science can be used to inform the intervention design, implementation and evaluation of a distance learning, social emotional learning intervention for VYAs.

**Methods:** This study will be delivered to 500 VYAs in Temeke District, Dar es salaam. Study participants will watch culturally-relevant, animated videos on social emotional mindsets and skills and content will be paired with experiential learning activities over a period of 10 weeks. A nested smart-phone based study will practice learning social emotional skills and mindsets through engagement with multi-media material via the WhatsApp messenger application. Surveys and in-depth interviews will be administered to adolescents, their parents/caregivers and teachers before and after the intervention to evaluate the effect of the intervention on study outcomes.

**Discussion:** This study is among the first to provide results on how to effectively design a distance-learning intervention to promote social emotional learning and identity development within a low-resource context. The findings will provide substantial evidence to inform new intervention approaches that are effective in low-resource contexts and strategies to reach scale among similar programs invested in leveraging technology to support adolescent health and development.

**Clinical Trial registration:** Study registered with ClinicalTrials.gov. Identifier number NCT0445807.

## Introduction

The Lancet Youth Commission Report highlighted the need for investment in the largest generation of 10- to 24-year-olds in human history (1). By 2018, there were ~1.24 billion adolescents representing 16% of the global population (2). Research has often focused on older adolescents (15–19 years) and missed the opportunity to intervene earlier where targeted interventions may have a greater impact (3, 4). The rapid growth in social emotional learning and identity development during early adolescence begins during the onset of puberty. During this time, physical, cognitive and behavioral maturation presents unique opportunities for intervention. This rapid period of maturational changes is sensitive to social emotional learning and identity formation experiences (5). For example, early adolescence is a time when adolescents experience enhanced motivation to embrace novelty, master new learning, take risks, and seek social reward (6, 7). Plasticity of the adolescent brain allows for rapid and dynamic development that both shapes the ways that adolescents experience the world, and responds to these experiences through reorganization of neural circuitry involved in processing emotions, risks, rewards and social relationships (8, 9). These developmental changes can lead to lasting impacts over the lifespan.

In low-resource contexts, adolescents face additional stress and adverse life experiences. Findings from research suggest targeting very young adolescence rather than later adolescence, can better improve long-term health trajectories by attenuating risks for health that increase during later adolescence such as depression, anxiety, violence and forced sex (1, 10, 11). Further evidence suggests that interventions that target very young adolescents lower HIV/AIDS infection rates, decrease the number of unwanted pregnancies, and improve a variety of indicators of health and well-being (12–14). As a result, there has been an increasing focus in sub-Saharan Africa to target early adolescence to improve health trajectories and reduce gender-based health disparities (15). Tanzania is experiencing a surge in the youth population, with ~12.2 million children between the age of 7–17 years making up 20% of the total population. The total population in this age group is expected to double by 2055 (16).

Since the introduction of free primary education in 2001, Tanzania has made strides to improve access to education. Between 2004 and 2010, enrolment in secondary education tripled for girls and quadrupled for boys, and by 2012 over 8.7 million (71%) of children between 7 and 17 years were enrolled in school (17). While the introduction of free primary education has resulted in higher secondary education enrollment, it has also revealed important equity gaps. Only a third of children that start primary school complete the cycle in seven years. The transition rates largely favor boys, with ~21% of boys joining secondary schools compared to 16% of girls (17). The results of a child poverty study conducted in Tanzania found that 74% of children in Tanzania are affected by multidimensional poverty while 29% live in households below the monetary poverty line (18). These educational and economic challenges have important impacts on adolescent health and well-being in both the short and long-term.

In 2016, one in four adolescent girls aged 15–19 years had begun childbearing, reflecting a 4% increase in teenage pregnancy since 2010 (19). Unfavorable sexual and reproductive health outcomes for girls have been attributed to the fact that cultural norms in Tanzania often support inequitable gender norms, beliefs and behaviors, particularly around adolescent sexual and reproductive health and educational and employment expectations. Normative gender roles provide boys with more autonomy, freedom to engage in activities and employment outside of the home, whereas girls often have less autonomy and are encouraged to attend to home chores with limited encouragement to pursue career goals (20, 21). A cultural prototype of a chaste female student is highly valued. At the same time, female sexuality is perceived as a resource to meet financial needs which results in exploitation, and coerced, transactional sex. Disparities in educational enrollment patterns and outcomes have increased attention on addressing gender inequities during adolescent development to achieve positive health and well-being outcomes.

**Figure 1:**
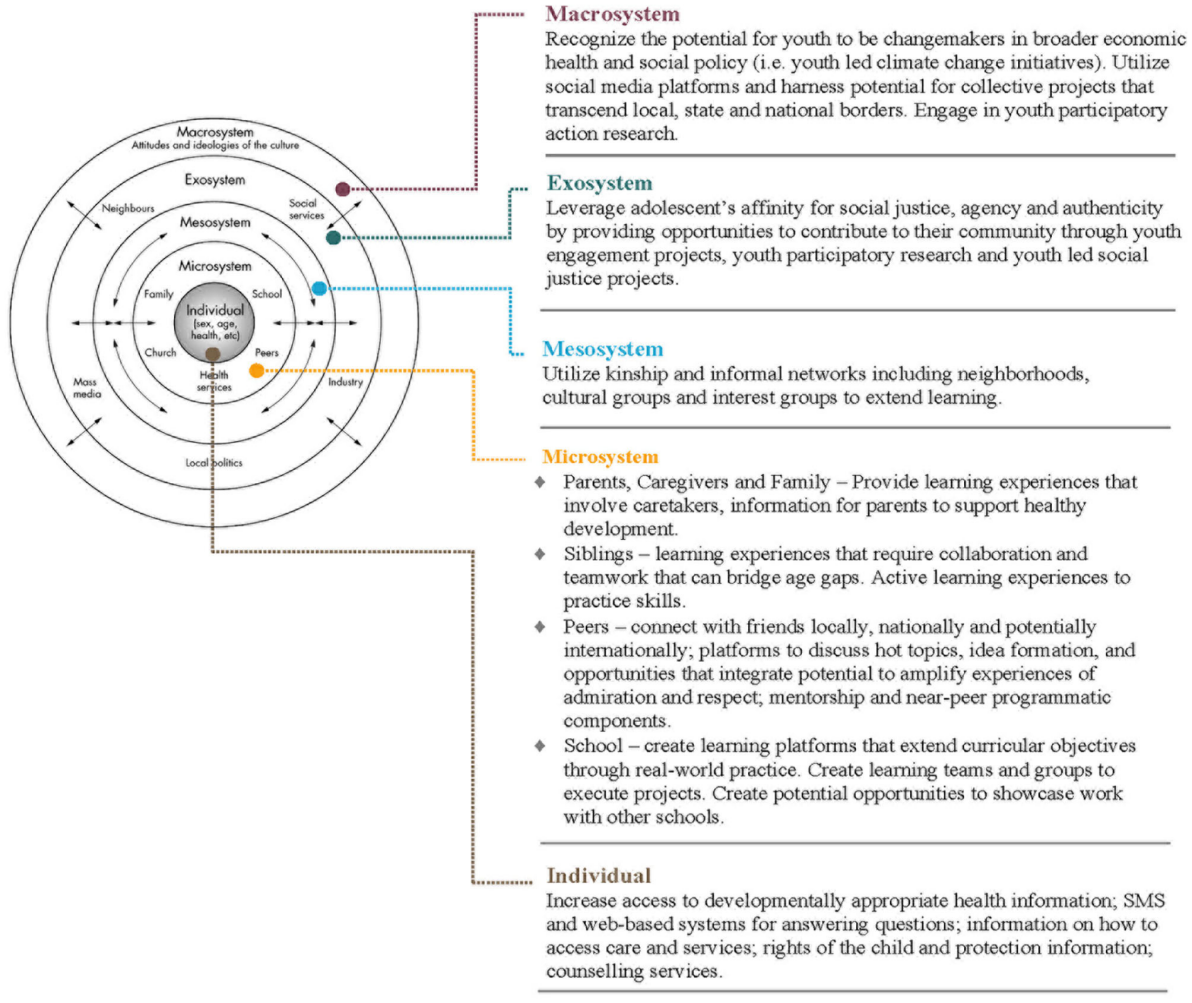
An ecological model of technology enabled learning opportunities for adolescents.

Delivering opportunities for social emotional learning experiences for very young adolescents using technology can enrich learning and be a flexible adaptation to complement learning in classrooms or to provide opportunities to continue learning when school-based learning is limited due to risks to health such as the COVID-19 pandemic. More recent research has focused on distance learning, as a modality to potentially replace in-person instruction (as has been required in many places due to COVID-19), and as a complement to traditional in-person education. Behavioral scientists underscore two mechanisms that motivate adolescents to use technology. First, rapid changes in adolescent's neurodevelopment motivate adolescents to seek autonomy, novel experiences, and exploration that can be scaffolded through use of digital technologies (22–24). Secondly, VYAs are increasingly invested in and learning about social relationships. Adolescents seek reward, respect and admiration from peers and are sensitive to feelings of rejection, disapproval and humiliation (25, 26). These experiences motivate adolescent social emotional learning and behavior. While peer relationships have greater importance along adolescent developmental trajectories, siblings, parents, extended family and the community also have the potential to enhance or diminish social emotional learning. An ecological model of adolescent learning is useful to consider opportunities for technology to leverage the sensitive window of early adolescent development across the individual, microsystem, mesosystem, exosystem and macrosystem (Figure 1). This research framework underscores opportunities to use technology to provide positive engagement with peers, family and the community in adolescent learning. Furthermore, use of technology within different contexts requires adaptation to ensure learning experiences are culturally resonant and tailored to very young adolescent in order to achieve effective intervention programming (22).

When the World Health Organization declared COVID-19 a global pandemic on 11th March 2020, many countries responded by enacting school-wide closures and/or distance learning alternatives that affected millions of children worldwide (27). How well schools and households are equipped to engage in remote learning differs greatly worldwide and within countries. In sub-Saharan Africa, penetration of mobile phone technology is high, with 80 subscriptions per 100 people, although only ~25% of these subscribers have access to internet services (28). In Tanzania, urban, younger, educated and English-speaking Tanzanians are most likely to use cell phones (29). While access to computers or tablets is limited, especially for young adolescents, the rapid uptake of technology provides new opportunities for learning (30). In 2016, the mobile operator Tigo Tanzania offered free WhatsApp access to its 10 million subscribers (31). Preliminary research with adolescents in Dar es Salaam and Mtwara indicates that adolescents ages 15–19 are increasingly using phones to access the internet (32). Adolescents are often among the earliest adopters of new technology and online learning platforms, however increased access to online information can accelerate both negative and positive developmental trajectories. Distance learning and technology can provide social emotional learning experiences that are designed to synergize with the rapid and dynamic changes in development that begin at the onset of puberty. These innovations include opportunities to enhance feelings of social reward and admiration, scaffolded learning that encourages risk taking, exploration and persistence, and, recognition of mastery of new skills.

Low-income countries face challenges to the effective utilization of technology including financial barriers to accessing technology, lack of regulation, long standing fear that technology will be used to access salacious content, and limited network connectivity. Amid these challenges, some promising remote learning programs are emerging. In Rwanda where radio reaches almost 99% of the population, lessons on primary-level literacy and numeracy are broadcasted daily for 20-min (33). Lessons are designed so that students can listen to content on their own, but parents and caregivers are encouraged to participate and support learning at home (34). One study in Turkey suggests that using WhatsApp Messenger application as a supplementary technology can increase the success of the students learning in the traditional classroom environment (35). Similarly, in Ghana, a program that uses WhatsApp Messenger to teach young learners found that a blended mobile learning structure that includes an intentional design and a step-by-step approach to teach both teachers and students can make communication easier and faster, including enhancing idea sharing among students (36).

WhatsApp is a free, internet-based messenger application that can be used to send multimedia messages like photos, videos, audio and simple text messages (37). Evidence shows that WhatsApp has the ability to increased student motivation and engagement around learning by using images to support information texts (35). In addition, this messaging service permits the unlimited and free sharing of multimedia. The WhatsApp platform is popular because it is highly interactive, allows timely feedback from instructors to the learner and permits attachments and sharing of classroom materials. Technology can also provide enhanced flexibility in instruction. For example, WhatsApp and SMS text-based systems can enhance or supplement learning access in more remote locations (38). These technology platforms appeal to adolescents because they provide both individual autonomy in learning and versatility in instructional content (39).

Unstructured Supplementary Services Data (USSD), is a session-based, real-time communication service that sends messages across a telecommunications network directly between a mobile client and an application server. USSD is similar to Short Message Service (SMS), however, USSD has a shorter turnaround response time, is cheaper and involves simple operations that can be assessed by older-model handset devices that are common in low-resource areas (40). Students interact with USSD by dialing a digit on the phone's keyboard in response to a question from the server. Messaging can be used between the mobile client and a pre-scripted program can to enhance interaction and support participatory learning. Other interventions have used different technologies such as tv and radio to reach students. In Sierra Leone an educational radio program (DevelopAfrica) was launched for students across the country, an initiative that was designed in response to the 2014 Ebola Outbreak (34).

Interactive technology can improve student engagement and has the potential to provide active, experiential learning that includes social interaction with peers. Platforms that facilitate social interaction are well-matched to the sensitive developmental window of early adolescence where adolescent neurodevelopment enhances motivation for social learning. Distance learning approaches offer opportunities to reach scale even in low-resource contexts. Investment in programs targeting early adolescence can leverage the sensitive window for learning that emerges with the onset of puberty through use of technology facilitated distance learning to improve adolescent health trajectories.

Our study follows the first intervention trial of the Discover Learning Intervention (*Discover Learning*), which aimed to support up to 500 very young adolescents and their families in Tanzania, with social emotional experiential learning content to support social emotional mindsets and skills that lead to healthy identity development. The intervention will be delivered over 10 weeks using a ten-module curriculum disseminated via distance learning and technology modalities. The specific target areas of adaptive social emotional mindsets and skills include: gender equity, teamwork, curiosity, growth mindset, persistence, purpose, utu, empathy and resilience, social responsibility and cooperation, and generosity. Learning materials are curated based on these ten social emotional mindsets and skills and will be translated into adolescent-friendly scripted videos aired to adolescents and their families via television and/or WhatsApp. Supplemental practice material includes a Parent/Caregiver-Youth workbook that will be disseminated to study participants and their families, with instructions to complete the activities individually or with near-peers, siblings, parents or extended family.

The first aim of *Discover Learning* is to provide a developmentally informed rationale for the design of the intervention and to detail the process related to the development, implementation and evaluation to enhance social emotional learning and identity development in very young adolescents. The primary research objective is to evaluate the extent to which a technology-based distance learning program can positively impact social emotional learning and identity development in very young adolescents. Our theory of change posits that if 10–14 year olds are exposed to information and material matched to the developmental window of very young adolescence, are engaged in activities that combine content and practice with their siblings/near peers, parents and community, there will be high impact improvements in outcomes for key social emotional skills and mindsets, enhanced equity in gender norms, beliefs and behaviors, and, health and well-being outcomes. These improved outcomes will help to set adolescents on early positive trajectories toward improved health, educational and economic outcomes. The results of this study will be used to: (1) determine the impact of *Discover Learning* on social emotional learning and identity development outcomes, (2) explore how technology can be used to amplify social emotional learning and identity development, and (3) examine how involvement of siblings/near peers, parents/caregivers and extended family can amplify study outcomes. *Discover Learning* is a strategic partnership between University of California Berkeley, Health for a Prosperous Nation (HPON), Camara Education and Ubongo Kids.

## Materials and Methods

### Study Setting

This study will be conducted in Dar es salaam, Tanzania. Tanzania currently has ~40.17 million sim card owners, representing 77% of the population (41). Dar es Salaam, which is the capital city of Tanzania, has five administrative districts. This research will be carried out in Temeke District, a peri-urban district. Study participants will be selected from four primary schools out of 114 schools within the district. The number of children enrolled in primary school in Temeke is 170,477; 49% are boys and 51% are girls. The average classroom size in Tanzania's primary schools is 80 students for each teacher (42). On 16 March 2020, the Ministry of Health of Tanzania announced the first case of COVID-19 (43). This announcement was rapidly followed by a directive to close schools indefinitely. In addition, measures such as prohibition of public gatherings and closure of recreational centers were established (44). Schools later reopened after 3 months of closure.

### Study Design

This protocol describes a study that will be conducted to test the impact of *Discover Learning*, a social emotional learning and identity development intervention for very young adolescents. The study employs an intervention design that leverages the principles of adolescent developmental science to improve early precursors of health inequities. A sample of 10–14 year old adolescents will be selected from four primary schools located in peri-urban areas of Temeke District in Dar es Salaam. Participants enrolled in *Discover Learning* will receive a distance learning intervention or will participate in a nested sub-group that includes additional smartphone-based content.

#### Intervention

Participants will be invited to watch ten episodes of video content on specific target areas of adaptive social emotional mindsets and skill (gender equity, teamwork, curiosity, growth mindset, persistence, purpose, utu, empathy & resilience, social responsibility & cooperation, and generosity) designed specifically for very young adolescents in Tanzania. These videos are produced by Ubongo Kids, a Tanzania-based organization that develops engaging and locally relevant digital content for children in Africa. Ubongo Kids' *edutainment* (a combination of educational and entertainment content) is disseminated via TV and radio episodes to over 6.4 million East African households weekly. Episodes developed for this study will be aired on national television channel, Tanzania Broadcasting Corporation (TBC) every Saturday. Repeat episodes for each social emotional mindset and skill episode will be aired every Wednesday of each week. Episodes will run for an average of 15–10 min each. *Discover Learning* participants will be provided paper-form worksheets that will include: (1) individual activities, (2) peer/sibling activities, and (3) parent/caregiver activities that complement the Ubongo Kids episodes. Participants will be asked to complete short experiential learning activities individually, or with a peer/sibling or adult after viewing the corresponding Ubongo Kids episode. Reflective learning responses to activities will be submitted via paper-based worksheets to *Discover Learning* research assistants. In order to provide motivation to complete the activities and adhere to the intervention content, participants will receive feedback on reflective responses through receipt of responses from program assistants to reinforce and clarify comprehension. Participants will also receive reward stickers pasted to participant worksheets to signify achievements. Parents/caregivers of participating adolescents will receive reminders to their phones through USSD on when to expect a new television or radio episode, and when reflective responses will be collected by research assistants. Midweek, participants and their families will be kept engaged through booster activities and short, informative public health messaging sent to their phones such as tips and practices on hygiene and self-care.

#### Smartphone-Based Intervention Group (Nested Study)

**Figure 2:**
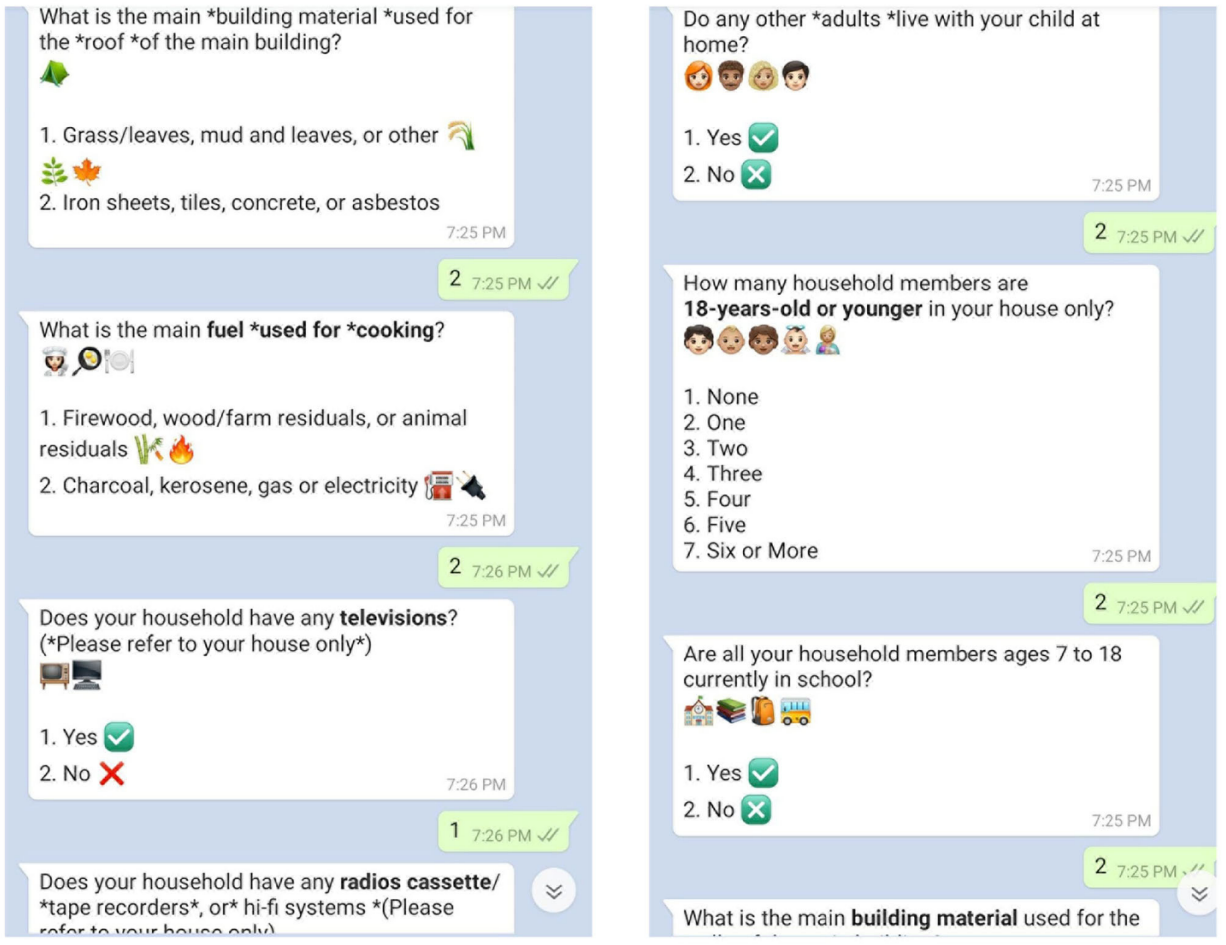
Screenshots of chat messages sent to participants in mobile-based group.

**Figure 3:**
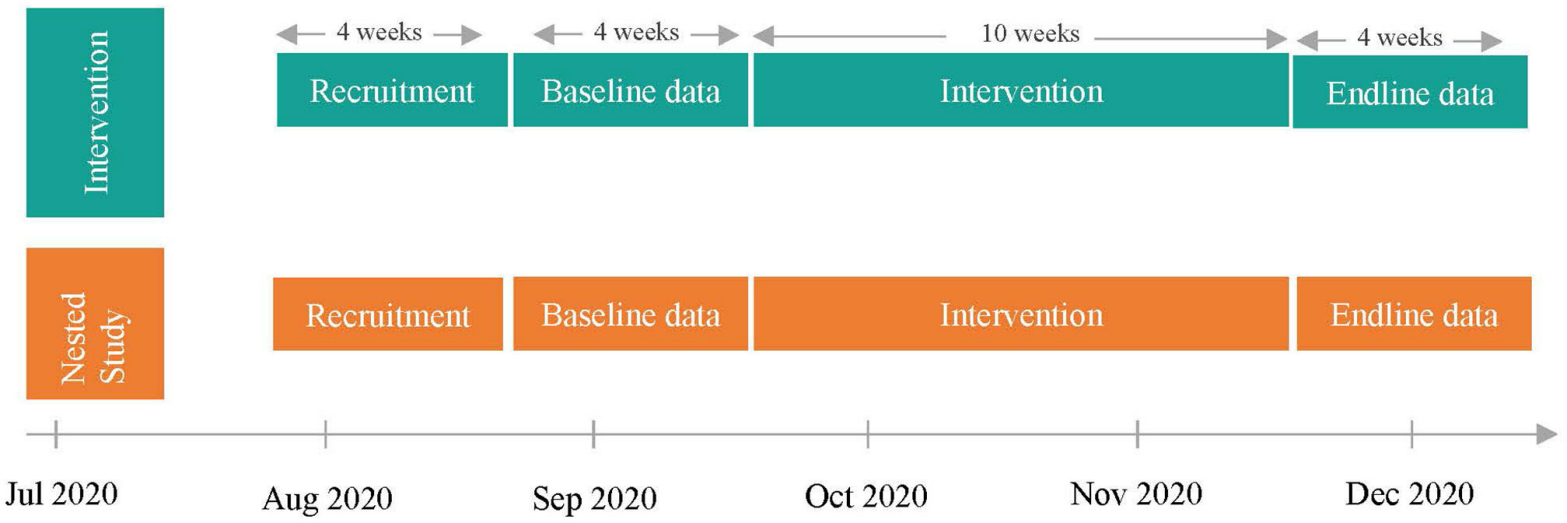
Timeline of study.

A nested study will be conducted with participants who have access to a smartphone and consistently use WhatsApp Messaging Application (WhatsApp), to assess the effectiveness of using mobile-based technology to implement the intervention. Sixty participants within the intervention group, and who use WhatsApp, will be invited to watch the same ten episodes of Ubongo Kids as the larger study, however, after each episode, experiential learning activities will be sent to this nested sample using WhatsApp instead of provision of paper-based worksheets. VYAs will be asked to complete these activities individually, with a peer/sibling or adult. Responses to activities will be submitted via pre-programmed short, quick chat sessions from the participants to the server as illustrated in Figure 2. Reflective responses will include multimedia material such as short audios, videos or pictures collected by VYAs. WhatsApp will enable youth to receive immediate feedback on their submissions via pre-programmed chat messages. Affirmative words and emojis/images such as congratulations, well done etc. will be sent to participants to reward completion of activities. Throughout the intervention, participants will receive reminders of upcoming episodes and short public health messages delivered via WhatsApp. Parent/caregiver-VYA dyads will receive honoraria in the form of internet data credits worth 1.72 USD upon completion of each week's activities. Figure 3 illustrates the study design, intervention components and anticipated timeline.

### Recruitment and Eligibility for Study

#### Youth

Study participants will include adolescents ages 10–14 recruited from four public primary schools in Temeke District, Dar es salaam. The study will leverage the relationship that the local implementation partner, Health for a Prosperous Nation has with the school district in order to gain access to the schools. The schools will be selected if they meet the following criteria: (1) have a population of more than 180 students aged 10–14 years, (2) socio-demographic status is typical of public schools in the area, (3) is a government-run public school, and (4) is not a boarding school. Schools will be excluded from the study if students attending the school have taken part in any behavior change intervention within the last 7 years and/or if the school has additional classes or programs after school. The eligibility criteria for study participants will include: (1) Must be a 10–14 years of age enrolled in 3rd to 6th grades in the four selected schools, (2) Adolescent has provided verbal assent to participate (3) Adolescent's parent/caregiver has provided written consent for participation in the study, (4) Parent/caregiver has access to a phone with USSD or WhatsApp capability (for the nested study); and (5) Adolescents must have access to television that broadcasts the TBC channel.

#### Parents/Caregivers

Parents or caregivers of adolescents participating in the intervention and who consent to participate in the study will be invited to complete a survey at baseline and endline. Parents and caregivers will agree to participate in the parent/caregiver-youth activities. Midweek, participating parents/caregivers and their families will be engaged through short, informative messaging sent to their phones such as tips and practices that support learning content and reminders on upcoming episodes.

### Sample

Based on the average population of each school, a cluster size of 100 will be required per school to detect a minimum detectable effect of 0.484 and a statistical power of 80% at a confidence level α = 0.05 for a two-tailed *t*-test (45). Assuming a participant dropout rate of 11.25% from the *Discover Learning* pilot study conducted in 2017 the final sample size will include 500 participants. The nested study will include all participants who have access to a smartphone with WhatsApp functionality. The study will be reported according to CONSORT guidelines.

### Study Instruments

This study will employ a mixed methods design to assess the impact of *Discover Learning* on participant social emotional mindsets and skills, gender norms, beliefs and behaviors and health and well-being outcomes. Quantitative data will be collected using sequential surveys administered to adolescents and their parents/caregivers at baseline and endline. Qualitative data will be collected at endline through in-depth interviews with adolescents and parents/caregivers, and key informant interviews with teachers. All surveys were pretested by trained researchers in the study setting.

#### Quantitative Data

##### Pre-Intervention Survey

The pre-intervention survey will include a 29-question survey to understand household social and demographic characteristics, phone usage, access to television and engagement with siblings/adults in the family. The survey will be delivered to all parents/caregivers of study participants. Surveys will be sent via USSD or WhatsApp and will take 10–15 min to complete. The results of the baseline survey will be to inform additional technological support required by study participants.

##### Baseline and Endline Survey

The adolescent survey will be administered at baseline and endline to measure changes in social emotional learning and health and well-being outcomes. The survey also seeks to capture changes in gender norms, beliefs and behaviors and positive attitudes toward technology as a learning tool. Measures are included to identify involvement of siblings, parents/caregivers and extended family in the intervention modules and seeks to identify which components of the intervention hold the greatest promise for innovation, scale, and adaptation. The survey questions are adapted from various validated measurement scales and modified to suit the study context and target group. One area of focus for this survey is the testing of a contextually relevant principle of *Utu*, an African philosophy of altruism, kindness, compassion and empathy. A summary of all measures included in the survey are listed in Table 1. The VYA survey will be administered by researchers via a phone call or sent via WhatsApp to participants in the nested study group, and will take 45–60 min to complete.

**Table 1:** Summary of measures to be tested in the *Discover*.

**Primary outcome**	**Validated measurement scale**
**Social Emotional Mindsets**	
Growth mindset	Yeager Growth Mindset (46)
Purpose	The Dimensions of Identity Development Scale (47)
Goal orientation	The Goal Orientation and Learning Strategies Survey (48)
Curiosity	The Trait State Curiosity Scale (49)
Persistence	The Scale for Measuring Persistence in Children (50)
Utu^1^	Measure Scale for Ubuntu (51); and Compassionate Engagement and Action Scale (52)
Social responsibility and cooperation	Teamwork and Collaboration Assessment for High School Students (53)
Persistence	The Scale for Measuring Persistence in Children (50)
Adverse life experiences	Adverse Childhood Experiences Questionnaire (ACE) (54)
**Social Emotional Skills**	
Teamwork	Teamwork and Collaboration Assessment for High School Students (53)
Empathy	The Empathy Questionnaire for Children and Adolescents (EmQue-CA) (55)
Gender equity	Gender Attitudes Scale - Choices curriculum (56)
Generosity	The Interpersonal Generosity Scale (57)
Resilience	Child & Youth Resilience Measure-Revised (CYRM-R) (58)
Coping skills	Spirito KIDCOPE checklist (59)

1 *Utu refers to is a traditional African philosophy, defined as communicating, caring, and sharing with humans in harmony with all of creation*.

##### Parent Survey

The parent survey contains questions on demographic socio-economic characteristics of households. Questions from the Simple Tanzania Poverty Scorecard (60) are adapted to determine how many participating households live below the national poverty line. A gender norm, beliefs and behaviors measure is included in the parent survey to capture changes in from baseline to endline in parents/caregivers. Evidence from both the pilot and first round of implementation of Discover Learning indicated that parental/caregiver gender norms, beliefs and behaviors did change after participation in Discover Learning. Including a measure of gender norms, beliefs and behaviors will allow the current study to capture these changes.

##### Discrete Choice Experiment (DCE)

A discrete choice experiment is included after completion of the VYA survey. The DCE provides adolescent participants with eight vignettes that represent different scenarios to elicit individual preferences and decision making pertaining to gender norms, beliefs and behaviors. The DCE will assess youth gender norms, beliefs and behaviors related to each of the ten learning modules. Following each vignette, participants choose from a set of cartoon images of either boys only, girls only, or boys and girls to respond to the illustrated vignette (61). All scenarios and images will be uploaded and presented digitally to participants.

#### Qualitative Data

Qualitative data will be collected in the form of in-depth interviews administered to participants, their parents/caregivers and teachers. Adolescents and their parents/caregivers will be invited to participate in a semi-structured in-depth interview after the completion of the intervention. Each interview will last no more than an hour and will provide information on respondent's experience with the intervention. Teachers will provide key-informant data to assess how well the intervention complemented in-person instruction.

### Data Collection

For this study, the data collection procedures will ensure minimal physical contact with study participants as a precaution against increasing the risk of transmission of COVID-19. Recruitment activities will start in July 2020. A copy of the announcement script for the study will be sent out to parents. The research team will call and screen parents prior to obtaining consent. If participants meet eligibility criteria, parents will provide verbal consent for their VYA to participate in the intervention. After receiving consent from parents/caregivers, verbal assent will be sought from the study participants to participate in *Discover Learning* and data collection activities. All assent/consent will be conducted in Swahili, the local dialect, by trained researchers in human-subjects research. Data from the pre-intervention and baseline youth and parent surveys will be collected via phone calls and WhatsApp in August and September 2020. Following the 10-week intervention, endline surveys will be administered to VYAs and parents/caregivers. In addition, researchers trained in qualitative methods will administer in-depth interviews over the phone to VYAs and parents/caregivers. All data collection activities are expected to be completed in January 2020. The datasets that will be generated and/or analyzed during this study will not be made publicly available due to the sensitive age of the study participants (10–14-year-old), but may be available from the corresponding author upon reasonable request. All approved IRB and data safety guidelines will be followed prior to providing data.

### Data Analysis

All quantitative data will be cleaned, coded and analyzed in Stata SE 16.0 (62). Statistical analysis will be conducted according to the Intention-To-Treat principle. Participants who do not complete all intervention modules due to attrition or who have missing data will be examined for inclusion in the final analysis. If <2% of the observations contain missing values on each variable and the pattern of missing values is random, then Listwise Deletion method will be used (63). If missing data is not determined to be missing at random, multiple imputation models will be used for analyzing adolescent data (64). All data will be examined for skewness or data non-normality. Multivariate regression models will be used to assess the effect of the intervention on study outcomes and will control for age, gender, socioeconomic status, school and household characteristics. Statistical significance for all analyses will be set at *p* < 0.05. Psychometric analysis of measures including exploratory and confirmatory factor analysis will be used to establish measure validity. Structural equation modeling (SEM) will be used to test structural paths between latent constructs. Potential mediating and modifying variables will be assessed. SEM models will be assessed for goodness of fit using indices including the root mean square error of approximation (RMSEA) (65), the comparative fit index (CFI) (66), the Tucker Lewis Index (TLI) (67) and the standardized root mean residual (SRMR). All qualitative data will be transcribed and translated verbatim from Swahili to English by trained translators. All participant identifiers will be removed from transcripts. All audio recordings of transcripts will be destroyed after transcription is completed and translation have been verified. Translations will be uploaded to Atlas.ti for analysis (68). A grounded theory approach will be used to identify emergent themes (69). The analytical steps will be conducted by two separate analysts. *Line-by-line coding of* each transcript will be used to identify initial codes that capture implicit meanings, and common relationships and significance between codes. Where appropriate codes will be left *in vivo* to preserve participant language and meaning. Next, *focused codes* will be developed from the most significant and/or frequent initial codes to make analytical sense of the data. *Axial coding* will be used to represent the content of focused codes and to connect relationships between codes, categories and concepts. During the application of focused and axial coding, analysts will meet to discuss and revise codes until consensus is reached. Researchers will write memos to capture connections and to construct analytic notes. Researchers will further hold discussions to help identify emergent themes and connections between themes through an iterative deductive process. Exemplar quotes will be selected to illustrate emergent themes. All transcripts will be stored in an access-controlled server that is located safely within the local implementation partner, HPON's Tanzania office and destroyed after 5 years.

## Discussion and Conclusion

This paper describes the protocol for *Discover Learning*, an intervention designed for very young adolescents to support social emotional learning and identity development using distance and technology-facilitated, experiential learning. Very young adolescence represents a population and a unique window of opportunity where investments in social emotional learning can lead to positive health trajectories and protect against a variety of health risks that emerge in later-adolescence. *Discover Learning* is unique because the intervention and evaluation design incorporated important insights from the developmental science of adolescence to enhance the effectiveness of social emotional learning during very young adolescence. Social emotional learning is not only important during early adolescence, but the mindsets and skills included in Discover Learning empower VYAs to cope with stressful life events in the proximal and distal future (70). Moreover, *Discover Learning* tests an innovative approach of delivering experiential social emotional learning through distance and technology enabled modalities in a low resource context. While other programs have been designed to include social and emotional learning to youth in low- and middle-income countries (71, 72), many of these programs are delivered in school and few include experiential activity learning that engages siblings and adults (73, 74). This study will make a novel and unique contribution to literature on strategies for delivering experiential distance-learning programs that engage siblings, parents/caregivers and extended family. Analysis from qualitative in-depth and key informant interviews will provide additional insight into the acceptability and integrity of the implementation strategy. These findings have the potential to be replicated in other low-resource contexts.

## Ethics Statement

The studies involving human participants were reviewed and approved by the UC Berkeley Committee for Protection of Human Subjects Institutional Review Board and the National Institute of Medical Research, Tanzania. Verbal informed consent and assent to participate in this study was provided by the participants' legal guardian/next of kin and adolescents, respectively, over the phone. This was done with respect to Covid-19 public health measures on social distancing and with directive from the institutional review boards.

## Author Contributions

The original *Discover Learning* study design has been conceived of and designed by MC, RD, CS, LR, and PN. Recruitment of participants, management, and project implementation will be performed by CS. Development of survey instruments and evaluation has been done by MC and SL. Manuscript preparation has been carried out by MC and SL. All authors have contributed critically and significantly to drafting a final manuscript and approved the final version.

## Conflict of Interest

The authors declare that the research was conducted in the absence of any commercial or financial relationships that could be construed as a potential conflict of interest.

## Abbreviations

CAB: Community Advisory Board
CFI: Comparative Fit Index
CONSORT: Consolidated Standards of Reporting Trials
DCE: Discrete Choice Experiment
HPON: Health for a Prosperous Nation
ICT: Information and Communication Technology
ITT: Intention-To-Treat
RCT: Randomized controlled Trial
RMSEA: Root Mean Square Error of Approximation (RMSEA)
SEM: Structural Equation Modeling
SMS: Short Message Service
SRMR: Standardized Root Mean Residual
TLI: Tucker-Lewis Index
UC Berkeley: University of California Berkeley
USSD: Unstructured Supplementary Services Data
VYAs: Very Young Adolescents.
